# Are lifestyle-factors in adolescence predictors for adult low back pain? A cross-sectional and prospective study of young twins

**DOI:** 10.1186/1471-2474-7-27

**Published:** 2006-03-15

**Authors:** Lise Hestbaek, Charlotte Leboeuf-Yde, Kirsten Ohm Kyvik

**Affiliations:** 1 The Back Research Center, Backcenter Funen, Part of Clinical Locomotion Science, University of Southern Denmark, Ringe, Denmark; 2 The Danish Twin Registry, University of Southern Denmark, Odense, Denmark

## Abstract

**Introduction:**

With more than half of the population experiencing low back pain (LBP) before the age of 20, research must focus on young populations. Lifestyle-factors might be important elements of prevention, since they are modifiable in nature. Therefore, the objective of the present study is to investigate the association between smoking, alcohol consumption and overweight in adolescence and 1) present LBP (cross-sectionally) and 2) the risk of future LBP (longitudinally).

**Methods:**

Data from 9,600 twins, aged 12–22, were analysed cross-sectionally with respect to associations between the above-mentioned lifestyle-factors and LBP. Eight years later, a follow-up survey (n = 6,554) was conducted and LBP at follow-up was correlated to the lifestyle-factors at baseline. Finally, the associations found to be significant were tested in a twin-control study design.

**Results:**

Our cross-sectional study demonstrated small, but statistically significant, positive associations between all three investigated life-style factors and LBP. In the longitudinal study, smoking at baseline showed a monotonic dose-response relationship with LBP at follow-up (OR up to 4.0 for those smoking >20 cig./day). There was also evidence of temporality (smoking preceding LBP). Adult LBP was negatively associated with adolescent alcohol consumption. We found no evidence of a dose-response relationship or temporality. There were no associations detected between adolescent overweight and adult LBP. In the twin-control study, the directions of associations were the same, but none of these associations attained statistical significance.

**Conclusion:**

Several of the Bradford Hill criteria for causality were fulfilled for smoking whereas the crucial aspect of temporality was missing for alcohol consumption and overweight. The twin-control study failed to confirm a statistically significant link between smoking and LBP.

## Background

Typical of the Western World, musculo-skeletal disorders in Denmark, of which LBP was the most common, comprised 50% of all work-related disorders in Denmark in the year 2000. In 2002, it was the second-most common reason for disability pension, being responsible for 22% of all disability pensions [1]. This indicates that interventions are currently – for the most part – introduced too late and are not effective enough [2,3]. Obviously, early intervention before the first onset of disease is the best method of prevention, but this requires a much better understanding of the nature of LBP than we have today. Etiology, origin of pain and natural course are important issues which are still only poorly understood. With more than half of the population experiencing LBP before the age of 20 [4], it is clear that if primary prophylaxis or interventions at first onset are to become a reality, researchers must focus on young populations.

The influence of various lifestyle-factors – such as smoking, alcohol consumption and overweight – is interesting from a public health perspective, because these are amenable to change. They are important factors when trying to comprehend the complex nature of LBP, and they might also provide valuable information in the search of high-risk populations. Previous studies of the link between LBP and smoking, alcohol consumption and overweight have been summed up in structured reviews [5-7]. They provide evidence of positive associations between LBP and all of these lifestyle factors, but possible causal links have not been established. When investigating this issue, it is important to study different age groups separately because the body responds to various stimuli in different ways throughout life [8]. It is likely that young people, in the phase of physical and mental development, are more susceptible to the influence of toxic substances, e.g. smoking and alcohol consumption. Likewise, it seems plausible that obesity during the growth phase will have a more detrimental anatomical and/or physiological impact than when the body is fully developed. Therefore, it is important to investigate the effect of these lifestyle factors in young populations and not merely extrapolate results, obtained in adult populations, to children and adolescents. Also, longitudinal studies are warranted if causality – rather than mere associations – is to be established. Therefore, we have conducted a mixed cross-sectional and longitudinal cohort study, based on a general, young population.

### Objectives

The purposes of the present study are to investigate the associations between smoking, alcohol consumption and overweight in adolescence and 1) present LBP (cross-sectionally) and 2) the risk of future LBP (longitudinally).

## Methods

### Study subjects

The Danish Twin Registry is one of the most comprehensive population based twin registers in the world, spanning a period of more than 125 years. The twins of interest for this study were born from 1972 to 1982. They were identified through the Danish Civil Registration System and represents 99% of twins born in that period. The twins can be regarded as representative of the general population since they have previously been shown to have the same mortality rate [9] and the same prevalence of various diseases as the population at large, e.g. insulin dependent diabetes [10], hand eczema [11], asthma and allergic rhinitis [12], and LBP [13]. Zygosity was determined by questions of similarity and mistaken identity, a method that has been shown to have a misclassification rate of <5% [14]. The database is described in detail elsewhere [15]. In 1994, a postal survey about general health, including past and present LBP, was conducted on 34,076 twins (96% of all), aged 12 to 41, who had previously agreed to participate in future studies. Only those born from 1972 to 1982, and thus aged 12 to 22 at baseline, were included in this study. Similar questionnaires were sent to the same population in 2002, when the participants in question were 20–30 years of age.

### Validation and reliability

The questions regarding LBP were modelled on the Nordic Back Pain questionnaire [16], which has been previously validated [17]. The reliability of LBP-questions from the 1994-survey has previously been considered to be satisfactory through the identification of logical errors [18]. Similarly, analyses of validity were performed on the data from the 2002-omnibus in a recent study, demonstrating only 0.3% illogical answers [19].

### Representativeness

Responders and non-responders at follow-up were compared with regard to age, gender, predictor- and outcome-variables at baseline.

### Variables

All variables used in this study were self-reported.

#### Outcome

The outcome-variable of interest for this paper was the number of days with LBP during the past year at baseline in 1994 and at follow-up in 2002. The exact wording of the question was: "How many days have you *altogether *had trouble with the lower part of your back during the past year?". This was accompanied by a drawing showing the lower back to cover the area from the 12^th ^ribs to the gluteal folds. This variable was analysed as either *persistent LBP*, defined as LBP for more than 30 days during the previous year, or as *LBP at all*, defined as LBP for at least one day during the previous year. Both of these are used for descriptive purposes. However, brief/transient episodes of LBP are very common and rarely influence the professional or the social life of the patient to any large extent. Persistent or recurrent LBP is a more interesting outcome variable, both from a socio-economic perspective and from the patients' perspective. Therefore, we chose persistent LBP as the outcome variable in the regression analyses.

#### Predictors

The relevant questions in relation to predictors were related to height, weight, smoking habits and alcohol consumption in 1994. The exact wording of these questions is available from the authors.

These variables were obtained as continuous variables. To estimate odds ratios and thus facilitate understanding of the results, the variables were transformed into binary and categorical variables. *Smoking *was defined as "yes/no" and as categories from 0 to >20 cigarettes per day, with increments of 10. *Alcohol consumption *was defined as units per day, where one unit is the equivalent of 12 grams pure alcohol. Like smoking, this was analysed both as a binary variable (yes/no) and as a categorical variable from <0.2 to > 1.0, with increments of 0.4 units. *Body mass index (BMI) *was calculated as weight in kilos divided by height in meters squared (kg/m^2^), and categorized into underweight, normal, overweight and obese. The cut point for underweight was 17, which defines the lowest 10% percentile, cut point for overweight was 24 and for obesity it was 29, which are average cut-point values for overweight and obesity, respectively, in the age group (12–22), based on internationally standardized cut-off points in relation to age [20]. Furthermore, BMI was also treated as a dichotomous variable: overweight (>24), yes/no.

### Statistical analyses

First, associations between the predictor variables (smoking, alcohol consumption and BMI) as binary variables and LBP were investigated individually in logistic regression analyses. Since LBP has often been shown to vary with age and sex, these and the following analyses were all adjusted for age and sex. The possibility of effect modifications/interactions between the predictor variables (smoking, alcohol consumption and overweight) was investigated by including the interaction-terms (multiplicative) in a multiple logistic regression analysis. The predictor variables demonstrating significant associations with LBP were included in a multivariate logistic regression analysis, including the statistically significant interaction-terms as well. This was done for LBP at baseline and LBP at follow-up. In order to illustrate the existence of a possible temporal relationship, this process was repeated for LBP at follow-up, including only the group that was symptom-free at baseline ('incidence cases').

Although the multivariate analyses adjust the estimates for the influence of age, sex etc, this might cover differences within strata of the various variables, therefore the estimates were explored with various stratifications: *1. Sex: *Stratification for sex was done to investigate the possibility of differences in susceptibility to those lifestyle-factors between boys and girls. *2. BMI: *Since the influence of toxic substances could be associated with size (smaller subjects are easier influenced due to higher concentrations of toxins per kilo, or the opposite: obese persons being more susceptible because of higher absorption-rates in adipose tissue) the multivariate analyses were also stratified for BMI-categories. *3. Age: *Finally, analyses were repeated stratified for age, due to the same rationale as for BMI.

Next, the possibility of a dose-response relationship was investigated through similar multivariate logistic regression analyses, this time using categorized predictor-variables. Again, all models were adjusted for age and sex.

Finally, a twin control study was conducted for each of the predictor variables. This involves the isolation of monozygotic twin pairs, discordant for LBP (i.e. only one in a pair with this condition), which were analysed as in a matched case-control study. The sample was too small for meaningful analyses of persistent LBP in this study-design. Therefore, LBP at all was used in this part of the study, both for the isolation of discordant twin pairs and as outcome variable. Since twins reared together can be assumed to be equal in most ways regarding external factors, and monozygotic twins furthermore have identical genes, this provides a very convincing case-control design. Again, odds ratios with 95% confidence intervals were calculated for the various predictor variables in relation to LBP.

All analyses were done using Stata 8.0 statistical software package. Robust inference, as provided by Stata's cluster option, was used to account for dependence of the data within twin pairs, and the significance level was set at 5%.

## Results

### Study sample and representativeness

The response rate at baseline was 84% (n = 9,608) and at follow-up it was 68% (n = 6,554). The study sample is described in Table 1 for both baseline and follow-up. There was a higher proportion of females at follow-up than in the baseline sample, otherwise there were no differences detected between responders at baseline and responders at follow-up. Table 2 shows the distribution of age, sex and the predictor variables in relation to LBP. This shows an increase in LBP-prevalence for the youngest age group from baseline to follow-up, and higher prevalence estimates for females than for males both at baseline and follow-up. It also gives an indication of a possible dose-response relationship between smoking and LBP as well as BMI and LBP.

### Single variable analyses

All three predictor variables had a statistically significant association with at least one of the outcome variables. Thus, all three variables were included in the multivariate analyses. Smoking was positively associated with both prevalent and future LBP, whereas alcohol consumption was positively associated with present LBP and negatively with future LBP. Overweight was associated with present LBP only.

### Interactions

There were no signs of interactions between the various predictor variables. Thus, none of the interaction-terms was included in the multivariate analyses. Data are not presented but are available from the authors.

### Multivariate analysis

As described above, all predictor-variables but no interaction-terms were kept in the model. All three predictor variables had a statistically significant positive association with present LBP, but only smoking was positively associated with future LBP. There was a statistically significant negative association between alcohol consumption at baseline and LBP at follow-up. No other associations were detected. The analyses of incidence cases in 2002 gave similar results to those from the whole sample. However, the negative association between alcohol consumption in 1994 and LBP in 2002 lost its statistical significance. The results of the multivariate analyses, using dichotomized predictor variables, are shown in 3.

### Stratifications

#### Sex

When stratified for sex, the estimated association between present LBP and alcohol consumption was stronger in boys than in girls (2.6 and 1.4, respectively) although the difference did not attain statistical significance. On the other hand, the association between overweight and present LBP was statistically significant, though not large (1.7) for girls, but non-significant for boys. As predictors, neither alcohol consumption nor BMI, were found to be significant, regardless of sex. The associations between smoking and LBP were similar for boys and girls.

#### BMI

Stratifying for BMI revealed no trend with increasing BMI, neither regarding associations between alcohol consumption and LBP nor between smoking and LBP, at baseline. However, at follow-up, odds ratios for LBP for smokers in relation to non-smokers increased with increasing BMI (1.5 / 1.6 / 2.6 / 11.3).

#### Age

The stratification for age revealed no differences in the estimates between the groups for the influence of smoking or BMI, and no trends were detected. However, the positive association between alcohol consumption and baseline LBP was stronger for the youngest group (12–15 y.o.a.) than for the oldest group (20–22 y.o.a.) although this was not statistically significant (odds ratio of 2.0 (1.0–4.1) for the youngest compared to 1.2 (0.8–1.9) for the oldest).

### Dose-response analysis

The dose-response analyses showed patterns of a monotonic dose-response for the cross-sectional associations for smoking and alcohol consumption but not for BMI. This is most evident for the associations for smoking. There was clear evidence of a monotonic increase in the risk of future LBP with increasing amount of cigarettes and also indications of a dose-response relationship between adolescent BMI and adult LBP. There were no signs of dose-response relationships between alcohol consumption and future LBP. Analyses of those without LBP at baseline ('incidence cases') gave similar results. The results relating to persistent LBP are shown in Table 4.

### Twin-control study

There were a total of 413 monozygotic twin pairs of which one had LBP at all during 1994 and the other did not. There was a statistically significant difference in the gender-distribution compared to the whole sample (56% (53–59) females in the MZ-sample and 51% (50–52) in the full cohort). Furthermore, the prevalence rates of smokers, alcohol consumers and overweight subjects were slightly higher than in the cohort at large. However, none of these differences were statistically significant. The results of the cross-sectional twin control study are shown in 5. This shows the odds ratio of being a smoker, an alcohol consumer or overweight for the twin with LBP at all compared to the LBP-free twin.

Result of the longitudinal twin control study is shown in 6. This shows the odds ratio of being a smoker, an alcohol consumer or overweight at baseline for the twin with LBP at all at follow-up compared to the LBP-free twin.

Due to the reduction of the sample to include only monozygotic twins, discordant for LBP at all, there was insufficient power to demonstrate significance of associations as weak as those in question. However, the pattern is the same as seen in the main analyses: all three lifestyle factors are positively associated with LBP in adolescence, whereas the association with adult LBP is positive for smoking, negative for alcohol consumption and close to none for overweight.

## Discussion

We found evidence of positive associations between the three lifestyle factors studied and persistent LBP in the cross-sectional part of the study. In the prospective part of the study, we found that persistent LBP was positively associated with smoking, negatively associated with alcohol consumption, and had no association with BMI. Furthermore, there was evidence of a causal link between smoking and persistent LBP, but not between alcohol or overweight and persistent LBP. Since the 'protective effect' of alcohol consumption does not show any signs of a causal relationship, this might be a proxy for some underlying psychological or social background variable resulting in a lower prevalence of LBP.

Our study was based on a large, young cohort. The young age of the subjects made it possible to study the influence of lifestyle factors at a time when the impact of work-related factors must be considered to be very limited. This was an important strength of our study, since job function and lifestyle factors often are associated, e.g. people in physically demanding jobs also tend to smoke more. This often complicates the interpretation of findings of previous studies. Another major strength of the cohort is that they are twins. This provides an opportunity to perform a twin-control study which is very powerful to control for various confounding factors, including genetics.

All variables in this study were self-reported which gives a possibility for underreporting of the investigated lifestyle factors. However, this did probably not alter our conclusions, since such underreporting is more likely to weaken than to strengthen the estimated associations. Psycho-social factors are important aspects, which this study did not address. We did not have the opportunity to study such factors, but have no doubt as to their importance for the complicated relationship between lifestyle factors and health. We will investigate this issue further in future studies. Physical activity, during leisure as well as at work, is another important lifestyle factor that had to be ignored in this study due to lack of information. This issue has, however, been studied to a large extent and results of previous reports should be considered to obtain a full picture.

Obviously, in order to study a causal link, there should be a plausible explanation. In the cases of smoking, alcohol consumption and overweight, we believe that such explanations exist. Reduced oxygenation (smoking), increased risk of injuries (alcohol consumption), and excessive wear and tear (obesity) are some plausible physiological explanations. However, according to Bradford Hill, there are also several other criteria that should be fulfilled before it would be possible to assume causality [21]. Our study was based on some of these.

The first criterion is that the association between the presumed cause and the outcome should be strong. Although we did find cross-sectional statistically significant associations between persistent LBP and smoking, alcohol consumption and BMI in a cohort of 9.600 12 to 22 year old Danes, none of these was strong (odds ratios from 1.3 to 1.9). We believed that a possible causal link between these life-style factors and LBP would emerge more clearly in this young study population, as there might be less competition from other causal factors such as the effects from ageing and occupational wear and tear than in adults. However, the strengths of associations in this population were not appreciably stronger than those reported in studies of adult populations [5-7] and they were similar to those of most other large studies (>5,000) on youngsters of the same age with regard to smoking [22-24] (The study by Kovacs et al being the exception, finding no association between smoking and LBP [25]). With regard to alcohol consumption and BMI, our results differ from those of others, who did not find any positive associations in young populations [23-25].

The second criterion is that a positive gradient, linking increased exposure to either a more severe disease or a higher prevalence, strengthens the indication for a causal link. In our study, most of the dose-response analyses did show a positive gradient, albeit generally weak for BMI and alcohol consumption. However, the odds ratios were now revealed as much stronger in the group of heavy smokers (odds ratios as high as 6.4). We know of only one study of children/adolescents in which the dose-response was reported, and they too found a positive gradient [23]. It is not known if others had the possibility to do these analyses, but failed to report their findings because they were negative.

The third criterion is that the exposure must precede the disorder. Eight years later, this was the case for smoking and LBP at all (odds ratio 1.4) and for smoking and persistent LBP (odds ratio 1.9). No such findings were noted for BMI or alcohol consumption, which seems to preclude a causal link for these two factors. We are aware of four studies from the last decade in which the aspect of temporality was reported, two of smoking [22,26] and two of BMI [27,28] and they all found LBP to follow exposure to the risk variables. In our study, the definition of an 'incidence' case was a person who reported no LBP the year prior to baseline. With a recall period of one year rather than total lifetime, this also included persons with LBP previous to the recall period. However, since long-term recall must be considered unreliable [19], better measures are not realistic.

The fourth criterion to consider is that positive findings should be consistent, which was the case in our study for smoking in that it was present in all of our analyses in relation to associations, dose-response and temporality. This was not the case for BMI and alcohol consumption.

The fifth criterion, that of reversibility, could not really be investigated in our study. The study subjects were not old enough to have smoked, overeaten and consumed alcohol for sufficiently long time to develop LBP and then to reverse their habit(s).

In addition, in our study we had the possibility to see how common the various potential risk factors were in genetically identical individuals who differed in relation to LBP. In other words, the prevalence rates of smoking, alcohol consumption and overweight at base-line were investigated in the 413 monozygotic twin pairs who were discordant on LBP at the same time. No significant associations were found, meaning that there is no obvious link between these life-style factors and LBP in genetically identical twins who lead very similar lives. This was also the case when looking at LBP at follow-up in relation to the life-style factors at base-line. There could be several reasons for this. Besides the most obvious explanation that the associations found in the main study are not real, but instead reflects some unknown confounders, the most plausible explanation is 'over-matching'. For example, the co-twin to a non-smoking twin is more likely to be a 'light' than a 'heavy' smoker, and thus the difference in lifestyle might be too small to demonstrate a significant effect on health. Finally, it must be kept in mind that the outcome variable in the twin-control study (LBP at all) was weaker than in the main study (persistent LBP). In short, statistically significant results of the twin-control study could have confirmed the associations found in the main study. On the other hand, non-significant results do not necessarily contradict the results from the main study.

Studies of more specific subgroups of LBP are warranted, in order to scrutinize the issue of smoking and LBP in the young further. Also, it would be relevant to search for a confounding factor that follows closely, not only smoking but also the amount of smoking, which may be one underlying – but as yet unknown – cause of LBP.

## Conclusion

In conclusion, several of the Bradford Hill criteria for causality were fulfilled for smoking but the crucial aspect of temporality was missing for BMI and alcohol consumption. The twin control study failed to confirm a statistically significant link between smoking and LBP.

## Competing interests

The author(s) declare that they have no competing interests.

## Authors' contributions

LH and CL-Y conceived of the study, and participated in its design. LH made the statistical analyses and drafted the manuscript. KOK participated in the design of the study. All authors read and approved the final manuscript.

## Pre-publication history

The pre-publication history for this paper can be accessed here:



## Figures and Tables

**Table 1:** Description of the sample – the baseline (1994) compared to follow-up (2002).

	**Baseline-sample**	**Follow-up-sample**
Responders, n (%)	9,609 (84%)	6,554 (57% of the original sample, 68% of the baseline sample)
Age at baseline, mean (range)	17.26 (11–22)	17.38 (11–22)
Female/male, n (%)	4,940 (51%) / 4,653 (49%)	3,676 (56%) / 2,867 (44%)
LBP at all 1994 (LBP > 0 days past year), n (%)	3,109 (33%)	2,246 (35%)
Persistent LBP 1994 (LBP > 30 days past year), n (%)	588 (6%)	401 (6%)
Number of days with LBP 1994, mean (SD)	10.49 (38.62)	10.83 (39.04)
LBP at all 2002 (LBP > 0 days past year), n (%)	Not applicable	2,546 (39%)
Persistent LBP 2002 (LBP > 30 days past year), n (%)	Not applicable	636 (10%)
Number of days with LBP 2002, mean (SD)	Not applicable	17.42 (53.94)
Smokers at baseline (ever smoked), n (%)	1,960 (21%)	1,250 (19%)
Alcohol-consumers at baseline (> 0.2 g alcohol/day), n (%)	5,349 (66%)	3,818 (67%)
Overweight at baseline (BMI > 24), n (%)	963 (10%)	663 (10%)

**Table 2:** Description of the sample – predictor-variables in relation to LBP.

		Status 1994. Baseline/ follow-up.	LBP at all (LBP >0 days past year) 1994	Persistent LBP (LBP >30 days past year). 1994	LBP at all (LBP >0 days past year) 2002	Persistent LBP (LBP >30 days past year). 2002
Age	12–15	3,146 / 2,061	447 (14%)	58 (2%)	742 (36%)	197 (10%)
	16–19	3,497 / 2,422	1,303 (37%)	232 (7%)	918 (38%)	213 (9%)
	20–22	2,820 / 2,060	1,343 (48%)	298 (11%)	883 (43%)	226 (11%)

Sex	Female	4,873 / 3,676	1,761 (36)%	366 (8%)	1,544 (42%)	424 (12%)
	Male	4,590 / 2,867	1,332 (29%)	222 (5%)	999 (35%)	212 (7%)

Smoking	0	85% / 86%	2346 (30%)	385 (5%)	2,080 (37%)	490 (9%)
	1–10	7% / 7 %	306 (46%)	69 (10%)	195 (44%)	57 (13%)
	11–20	8% / 7%	365 (72%)	107 (15%)	217 (49%)	73 (17%)
	>20	0% / 0%	33 (72%)	9 (30%)	13 (48%)	3 (11%)

Alcohol consumption	<0.2	56% / 54%	1084 (24%)	185 (4%)	1181 (38%)	322 (10%)
	0.2–0.6	23% / 24%	793 (43%)	157 (9%)	547 (40%)	130 (9%)
	0.6–1.0	11% / 11%	419 (46%)	81 (9%)	257 (39%)	55 (8%)
	>1.0	10% / 10%	389 (48%)	84 (10%)	238 (42%)	54 (10%)

BMI	<17	12% / 12%	163 (15%)	28 (3%)	262 (35%)	74 (10%)
	18–24	78% / 78%	2,459 (34%)	455 (6%)	1,964 (39%)	479 (10%)
	25–29	9% / 9%	376 (45%)	85 (10%)	242 (42%)	59 (10%)
	>29	1% / 1%	58 (49%)	12 (10%)	42 (47%)	11 (12%)

**Table 3:** Multivariate logistic regression analyses including age, sex and binary data of the three investigated predictor variables. Odds ratios with 95% confidence intervals for LBP. Statistically significant findings indicated in bold:

	1994 LBP >30 days past year. n = 588	2002 LBP >30 days past year. n = 636	2002 LBP > 30 days past year in incidence cases* n = 314
Smoking n = 1,960 Ref: non-smokers	**1.77 (1.44–2.17)**	**1.69 (1.36–2.11)**	**1.88 (1.32–2.69)**
Alcohol consumption n = 5,349 Ref: Non-drinkers	**1.66 (1.20–2.31)**	**0.74 (0.58–0.94)**	0.87 (0.61–1.23)
Overweight n = 963 Ref: BMI<24	**1.38 (1.06–1.79)**	1.12 (0.84–1.49)	1.43 (0.90–1.01)

* No LBP in 1994.

**Table 4:** Dose-response analysis of LBP in relation the categorical predictor variables based on multivariate logistic regression including all variables (age, sex, smoking, alcohol consumption and BMI). Odds ratios with 95% confidence intervals for LBP. Statistically significant findings indicated in bold.

		1994 LBP >30 days past year. n = 588	2002 LBP >30 days past year. n = 636	2002 OR for LBP > 30 days past year in incidence cases*. n = 314
Smoking	0 cig./day	1.00	1.00	1.00
	1–10 cig./day	**1.38 (1.02–1.88)**	**1.60 (1.16–2.20)**	**1.76 (1.10–2.82)**
	11–20 cig./day	**2.00 (1.53–2.63)**	**2.13 (1.57–2.88)**	**2.12 (1.30–3.46)**
	>20 cig./day	**6.38 (2.66–15.35)**	**4.00 (1.11–14.48)**	**-**
				
Alcohol consumption	<0.2 units/day	1.00	1.00	1.00
	0.2–0.6 units/day	**1.29 (1.00–1.65)**	**0.76 (0.59–0.97)**	0.82 (0.57–1.18)
	0.6–1.0 units/day	**1.41 (1.02–1.93)**	**0.71 (0.51–0.98)**	0.82 (0.49–1.38)
	> 1.0 units/day	**1.58 (1.14–2.19)**	0.85 (0.60–1.20)	1.25 (0.76–2.06)

BMI	< 17 kg/m^2^	1.00	1.00	1.00
	17–23.9 kg/m^2^	1.00 (0.62–1.59)	1.00 (0.73–1.38)	0.91 (0.65–1.29)
	24–28.9 kg/m^2^	1.41 (0.82–2.43)	1.10 (0.72–1.69)	1.21 (0.70–2.09)
	= 29 kg/m^2^	1.01 (0.41–2.49)	1.26 (0.61–2.61)	1.33 (0.45–3.94)

* No LBP in 1994.

**Table 5:** Twin-control study. Odds ratios for the twin with LBP in 1994 of being exposed to the investigated risk factor in that same year. Based on monozygotic twin pairs, discordant for LBP at all in 1994 (413 pairs).

	The twin without LBP in 1994	The twin with LBP in 1994
Smoking at baseline OR (95%CI)	1.00	1.26 (0.70–2.28)
Alcohol consumption at baseline OR (95%CI)	1.00	1.79 (0.89–3.72)
Overweight at baseline OR (95%CI)	1.00	1.75 (0.82–3.90)

**Table 6:** Twin-control study. Odds ratios for the twin with LBP in 2002 of having been exposed to the investigated risk factor in 1994. Based on monozygotic twin pairs, discordant for LBP at all in 2002 (334 pairs).

	The twin without LBP in 2002	The twin with LBP in 2002
Smoking at baseline OR (95%CI)	1.00	1.50 (0.73–3.19)
Alcohol consumption at baseline OR (95%CI)	1.00	0.58 (0.19–1.61)
Overweight at baseline OR (95%CI)	1.00	0.89 (0.30–2.60)

* No LBP in 1994.

## References

[B1] Biering-Sørensen F, Kjøller M (2004). Musculo-skeletal diseases. Ugeskrift for læger.

[B2] van den Hoogen HJ, Koes BW, van Erijk JT, Bouter LM, Deville W (1998). On the course of low back pain in general practice. A one-year follow-up study. Ann Rheum Dis.

[B3] Leino PI, Hänninen V (1995). Psychosocial factors at work in relation to back and limb disorders. Scand J Work Environ Health.

[B4] Leboeuf-Yde C, Kyvik KO (1997). At what age does low back pain become a common problem?. Spine.

[B5] Leboeuf-Yde C (1999). Smoking and low back pain. A systematic literature review of 41 journal articles reporting 47 epidemiologic studies. Spine.

[B6] Leboeuf-Yde C (2000). Alcohol and low-back pain: A systematic literature review. J Manipulative Physiol Ther.

[B7] Lebeouf-Yde C (2000). Body weight and low back pain. A systematic literature review of 56 journal aricles reporting on 65 epidemiological studies. Spine.

[B8] Bortz WM (2005). Biological basis of determinants of health. Am J Public Health.

[B9] Christensen K, Vaupel J, Holm NV, Yashin A (1995). Twin mortality after age 6: fetal origins hypothesis versus twin method. BMJ.

[B10] Kyvik KO, Green A, Beck-Nielsen H (1995). Concordance rates of insulin dependent diabetes melitus: A populationbased study of young Danish twins. BMJ.

[B11] Bryld LE, Agner T, Kyvik KO, Brondsted L, Hindsberger C, Menne T (2000). Hand eczema in twins: a questionnaire investigation. Br J Dermatology.

[B12] Skadhauge LR (1999). Genetic and environmental influence on asthma. A populationbased study of Danish twins.

[B13] Hestbaek L (2003). The natural course of low back pain and early identification of high-risk populations.

[B14] Hauge M (1981). The Danish Twin Register.

[B15] Kyvik KO, Christensen K, Skytthe A, Harvald B, Holm NV (1996). The Danish Twin Register. Dan Med Bull.

[B16] Kuorinka I, Jonsson B, Kilbom A, Vinterberg H, Biering-Sørensen F, Andersson G, Jørgensen J (1987). Standardized Nordic questionnaires for the analysis of musculoskeletal symptoms. Applied Ergonomics.

[B17] Biering-Soerensen F, Hilden J (1984). Reproducibility of the history of back trouble. Spine.

[B18] Hestbæk L, Leboeuf-Yde C, Kyvik KO, Vach W, Russel MB, Skadhauge L, Svendsen A, Manniche C (2004). Comorbidity with low back pain. A cross-sectional population-based survey of 12–22 year olds. Spine.

[B19] Hestbaek L, Leboeuf-Yde C, Kyvik KO, Manniche C Course of low back pain from adolescence to adulthood. Eight years follow-up. Spine.

[B20] Cole TJ, Bellizzi MC, Flegal KM, Dietz WH (2000). Establishing a standard definition for child overweight and obesity worldwide: international survey. BMJ.

[B21] Hill AB (1965). The environment and disease: Association or causation?. Proc R Soc Med.

[B22] Power C, Frank J, Hertzman C, Schierhout G, LiL (2001). Predictors of low back pain onset in a prospective British study. Am J Public Health.

[B23] Vikat A, Rimpea M, Salminen JJ, Rimpela A, Savolainen A, Vintanen SM (2000). Neck or shouderpain and low back pain in Finnish adolescents. Scand J Public Health.

[B24] Ghandour RM, Overpeck MD, Huang ZJ, Kogan MD, Scheidt PC (2004). Headache, stomachache, bachache, and morning fatigue among adolescents girls in the United States. Arch Pediatr Adolesc Med.

[B25] Kovacs FM, Gestoso A, Gil del Real MT, Lopez J, Mufraggi N, Méndez JI (2003). Risk factors for non-specific low back pain in schoolchildren and their parents: a population based study. Pain.

[B26] Feldman DE, Rossignol M, Shrier I, Abenhaim L (1999). Smoking. A risk factor for development of low back pain in adolescents. Spine.

[B27] Lake JK, Power C, Cole TJ (2000). Back pain and obesity in the 1958 British birth cohort: cause or effect?. J Clin Epidemiol.

[B28] Peltonen M, Lindroos AK, Torgerson JS (2003). Musculoskeletal pain in the obese: a comparison with a general population and long-term changes after conventional and surgical obesity treatment. Pain.

